# Development and rapid evaluation of services to support the physical health of people using psychiatric inpatient units during the COVID-19 pandemic: study protocol

**DOI:** 10.1186/s43058-021-00113-0

**Published:** 2021-02-03

**Authors:** Julie Williams, Elli Fairbairn, Ray McGrath, Amy Clark, Andy Healey, Ioannis Bakolis, Fiona Gaughran, Euan Sadler, Zarnie Khadjesari, Nick Sevdalis, IMPHS group, Kate Lillywhite, Isabel McMullen, Prashanth Reddy, Gavin Shields

**Affiliations:** 1 Centre for Implementation Science, Health Service and Population Research Department, Institute of Psychiatry, Psychology and Neuroscience, King’s College London, London, UK; 2 South London and Maudsley NHS Foundation Trust, London, UK; 3 King’s Health Partners Mind and Body Programme, Ground Floor, Counting House, Guy’s Hospital St Thomas St, London, UK; 4 Kings Health Economics, Health Service and Population Research Department, Institute of Psychiatry, Psychology and Neuroscience, King’s College London, London, UK; 5 Department of Biostatistics and Health Informatics, Institute of Psychiatry, Psychology and Neuroscience, King’s College London, London, UK; 6 Psychosis Studies, Institute of Psychiatry, Psychology and Neuroscience, King’s College London, London, UK; 7 National Psychosis Service, South London and Maudsley NHS Foundation Trust, London, UK; 8 Department of Nursing, Midwifery and Health, School of Health Sciences, Faculty of Environmental and Life Sciences, University of Southampton, Southampton, UK; 9 Behavioural and Implementation Science (BIS) research group, School of Health Sciences, University of East Anglia, Norwich Research Park, Norwich, UK

**Keywords:** Serious mental illness, Integration, Physical health, Implementation, Effectiveness, Cost-effectiveness, Evaluation

## Abstract

**Background:**

People diagnosed with a serious mental illness have worse physical health and lower life expectancy than the general population. Integration of mental and physical health services is seen as one service development that could better support this. This protocol describes the evaluation of the provision of a Virtual Physical Health Clinic (VPHC) and Consultant Connect (CC) services to one UK-based mental health Trust.

**Methods:**

Prospective, formative, pragmatic evaluation using both quantitative and qualitative techniques and driven by implementation science theoretical frameworks. The VPHC and CC are described along with the methodology being used to rapidly evaluate their implementation, effectiveness and potential economic impact in order to inform future roll out. We will assess the implementation process through quantitative data on uptake and reach and through self-reported data to be collected from interviews and the use of validated implementation outcome assessment measures. We will assess implementation strategies using the Expert Recommendations for Implementing Change (ERIC) strategies as a framework. We will assess the health economic impact of both services using established health economic methods including cost comparison scenarios and health service utilisation analyses.

**Discussion:**

Supporting the physical health management of people in psychiatric inpatient units is important in improving the physical health of this population. Integration of mental and physical health can help this to happen effectively. This initiative provides one of the first service evaluation protocols of its kind to be reported in the UK at the time of the COVID-19 pandemic.

Contributions to the literature

- The study advances the literature in understanding how physical and mental health services can be integrated to improve the physical health of people using mental health services.
- Study findings will contribute to our understanding of the implementation challenges of providing such an initiative.
- This study includes a comprehensive economic evaluation which will demonstrate how this can be done to provide importance economic data.

## Background

People diagnosed with a serious mental illness (SMI) such as schizophrenia and bipolar disorder generally have worse physical health and reduced life expectancy compared to the general population [1]. International studies have found the mortality gap to be between 10 and 20 years [2]. Recent literature has attempted to map the scale of this problem and understand its causes [3]. Studies have highlighted risk factors for excess mortality at the levels of individual, such as the effect of lifestyle factors and medication and the severity of symptoms of mental illness, at the service level, such as lack of coordination of care, and at the society level, such as continued stigma that inhibits access to equitable care [4], and has made suggestions for strategies to improve processes and outcomes such as better integrating mental health physical health systems. In the current global epidemiological context, there is emerging evidence that the COVID-19 pandemic will exacerbate the treatment gap to further disadvantage people with SMI due to disruptions to services and an exacerbation of inequalities in treating physical health problems [5–7].

The National Institute of Health and Care Excellence (NICE) have developed guidance for the management of physical health for people with schizophrenia and psychosis [8], but there is evidence that this guidance is not always followed [9]. There are few published reports of projects to improve physical health management on psychiatric units in the UK [10].

### The ‘integrating our mental and physical healthcare systems’ (IMPHS) project

The Integrating our Mental and Physical Healthcare Systems (IMPHS) project is funded by the Maudsley Charity and is working with the South London and Maudsley NHS Foundation Trust (SLaM) in London, UK, to seek to address risk factors for poor physical health outcomes for SLaM service users. IMPHS is part of the King’s Health Partners’ (KHP) Mind and Body programme which seeks to join up mental and physical healthcare and provide training and research to improve physical health outcomes for people with SMI. King’s Health Partners is a partnership between SLaM, King’s College London university and two local acute trusts: King’s College London NHS Foundation Trust and Guy’s and St Thomas NHS Foundation Trust.

The IMPHS project was planning services to support the management of physical health in the SLaM adult acute units before the COVID-19 pandemic. This paper describes the rapid development and introduction of adapted services to assist healthcare staff in SLaM to manage the physical health of patients in inpatient units during the COVID-19 pandemic.

Significant change happened across KHP to address the COVID-19 pandemic, in the form of service reconfigurations, from March 2020 onwards. This led to collaboration between the acute Trusts and SLaM designed to support care delivery to SLaM patients and to reduce unnecessary pressures on the acute Trusts. These changes are ongoing due to the changing nature of the burden of COVID-19 and the need to provide optimal care in a rapidly changing environment.

This protocol focuses on the implementation and evaluation of two distinct but related service developments to inpatient units in SLaM during the COVID-19 pandemic: the Virtual Physical Health Clinic (VPHC) and Consultant Connect (CC).

The VPHC was originally designed as a traditional face to face clinic, but this became unviable due to the pandemic. The aims of the VPHC are to firstly improve the management of physical health for patients on SLaM inpatient units both for long-term conditions and acute conditions (including COVID-19 sequelae although not acute COVID-19) by improving access to generalist advice and virtual appointments for complex cases and secondly to reduce inappropriate referrals to, and attendances at, general medical services by providing advice and guidance on managing physical health issues.

The Consultant Connect (CC) service was intended as a complementary intervention to the VPHC and aims to improve care and treatment for physical health conditions and reduce inappropriate referrals and admissions of SLaM patients to acute general hospitals by providing telephone-based advice on specific queries. This service will now be introduced within SLaM, to offer SLaM clinicians working on inpatient units prompt access to a medical clinical support service, provided by specialist colleagues based at local Trusts.

#### Study aims

The primary aim of the evaluation is to understand the process of rapidly implementing the VPHC and CC. This formative evidence on the implementation of both services will ensure that implementation can be rapidly optimised and supported in the long-term.

The secondary aim of the evaluation is to establish the acceptability and feasibility of both the VPHC and CC.

The tertiary aims of the evaluation are to:

1. Undertake an exploratory analysis to inform future roll out and impact evaluation through establishing the likely patient benefit of the interventions and developing a logic model. Through monitoring the types of referrals and contacts with CC and VPHC, evidence will be gathered on the likely effect of the interventions. For example, the exploratory phase will enable testing of whether it is plausible to expect the interventions to improve the management of long-term conditions such as diabetes.
2. Conduct an economic evaluation including initial cost comparison to establish if the interventions are plausibly cost neutral or cost saving and to view this evidence in light of likely patient benefit (or whether VPHC and CC are likely to at least deliver equivalent patient outcomes compared to usual care), without evidence of any detrimental effects, and acceptability to staff.

Initial assessments will inform decision-making regarding the longer term (i.e. post-COVID-19 crisis response) implementation and sustainability of these interventions. We will also evaluate any inequalities in the use of the service by investigating referrals for different demographic groups.

## Methods/design

### Design

This is a prospective formative evaluation, which takes into account the evolving needs of services and the COVID-19 pressures on services and staff over time. The design can be changed depending on any service reconfigurations or other changes as the pandemic progresses.

### Setting

This project will take place in SLaM inpatient units. SLaM is the largest mental health NHS Foundation Trust in England which provides mental health treatment and care in both community and inpatient settings across four London boroughs. Adult inpatient services are all situated on four sites (Maudsley hospital, Lambeth hospital, Lewisham hospital and the Bethlem Royal hospital) PLUS one additional rehabilitation unit in Lewisham. The VPHC will be trialled initially on the Maudsley hospital site but with rollout to other SLaM sites subject to an assessment after nine months. CC will be offered initially to all inpatient services and may be extended to Community teams in the future.

### Participants

It is expected that the majority of staff using the service will be SLaM trainee doctors (called ‘residents’ in some systems) and consultants (called ‘attendings’ in some systems) reviewing physical health issues. However, the evaluation will also capture other staff members potentially accessing the services (e.g. ward nurses, pharmacists).

### Data sources

The evaluation will use (i) pseudonymised individual level observational data interrogated using the Maudsley Biomedical Research Centre (BRC) Clinical Record Interactive Research (CRIS) system. The CRIS system has been developed to allow researchers access to deanonymised data derived from SLaM clinical data (the electronic Patient Journey System (ePJS)). CRIS also has linkages between SLaM data and other health data such as Hospital Episodes Statistics (HES) which is routinely collected data on hospital admissions in the NHS [11] (ii) service-level data routinely collected by SLaM and (iii) data collected from clinicians. Data from clinicians will be collected remotely initially in line with COVID-19 pandemic guidelines and will include baseline and follow-up surveys of ward staff to identify changes in time spent undertaking administrative tasks associated with physical healthcare management. Face to face evaluations may be introduced depending on government and local guidelines on infection prevention and control and social distancing.

## The virtual physical health clinic and consultant connect

The two novel services are described in Table 1

**Table 1:** Descriptions of the Virtual Physical Health Clinic and Consultant Connect services of this evaluation

Virtual Physical Health Clinic	Consultant Connect
The service will offer: • Two sessions per week of consultant physician time and one session per week of ANP time (one session = half a day). • A referral system via the SLaM electronic patient journey system (ePJS) with triage of referrals undertaken by members of the research team throughout the week. • 5 Virtual clinic appointments offered throughout the week via MS Teams. • Telephone support (30 min daily) for ad hoc queries where staff are unclear what specialism they should consult or where the patient in question has a number of co-morbidities. • Email response to queries, where requested • Provide virtual education and training sessions in response to issues identified in clinic referrals.	• Telephone and photo advice service for doctors providing specialist medical advice from colleagues based in local acute Trusts. • Set up in 2015 and has been used since October 2017 in general practice in Southwark and Lambeth • Available 9 am–5 pm, Monday–Friday with a few exceptions • Access is now extended to SLaM inpatient services. • Can be accessed by phone or app • Over 60 adult and paediatric specialisms made available to SLaM

### Use of VPHC and CC

**Fig. 1:**
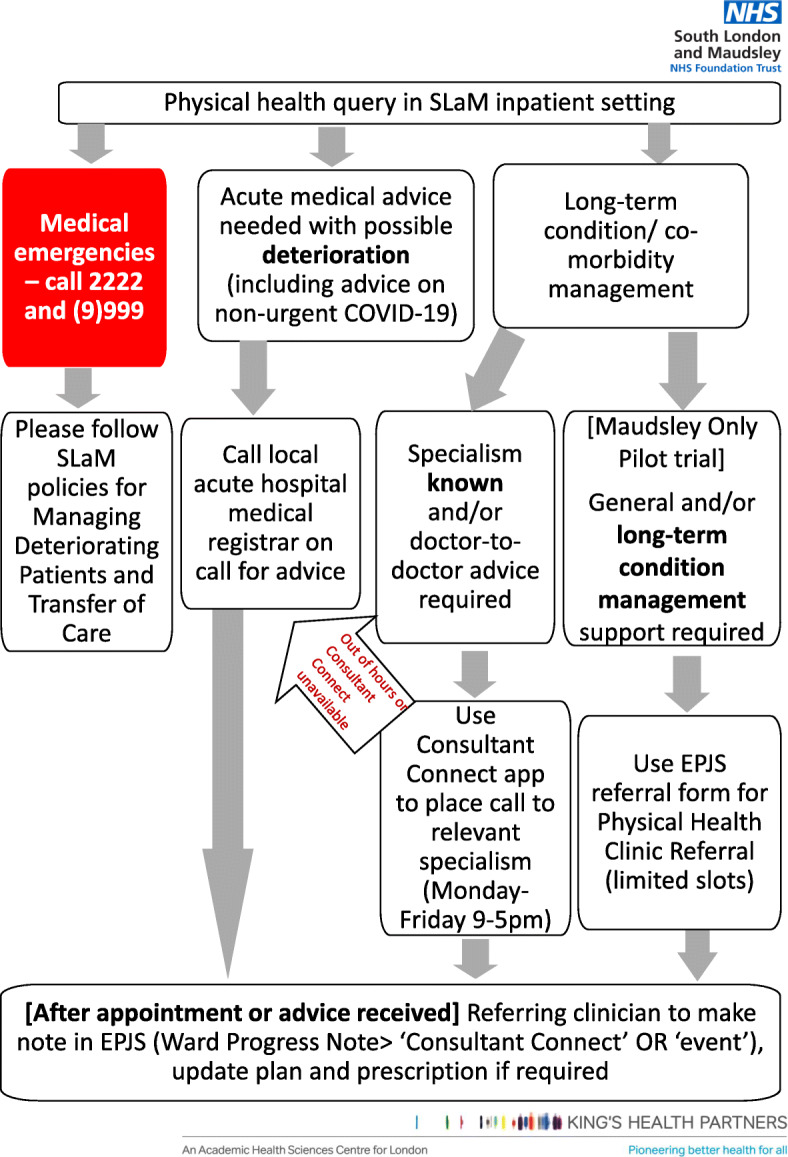
Physical health management flow chart

The VPHC and CC will be key to supporting SLaM staff to manage the medical needs of their inpatients. They provide different routes (see Fig. 1) for SLaM clinicians to access advice on the management of non-COVID-19-related physical health conditions, both chronic and acute. Each intervention offers support for clinicians with CC being appropriate for queries where referrers know the medical speciality they require support from and VPHC for queries where the speciality is not known or where the query is regarding management of non-urgent, long-term physical health conditions, multiple conditions or other complex presentations. Acute deteriorations of COVID-19 are managed through standard local pathways.

### Implementation of VPHC and CC in SLaM during COVID-19 pandemic

#### VPHC

VPHC is accessed using a brief referral form on the electronic healthcare record—ePJS—which will ask for details of the referrer and what the referral is being made for. Each referral request will be triaged by IMPHS project clinical staff and the King’s College Hospital (KCH) Consultant, and the referrer will be offered either a virtual appointment for the referrer (with patient present) or telephone advice for referrer only. Where appropriate to do so, referrals will be signposted onwards. The process will be documented and monitored by the IMPHS Team to ensure referrals are actioned in a timely manner. All appointments will be conducted using phone or Microsoft Teams software, providing a discussion between the KCH consultant, the referrer and the patient. The advice and discussion will be recorded on ePJS by the referring clinician.

The VPHC will be trialled on the Maudsley site initially to test the systems and ascertain the level of use. Having quantified the referral rate over the initial 9-month trial and if the system is working successfully, the VPHC will be rolled out across further SLaM adult inpatient sites subject to agreement of resource.

**Fig. 2:**
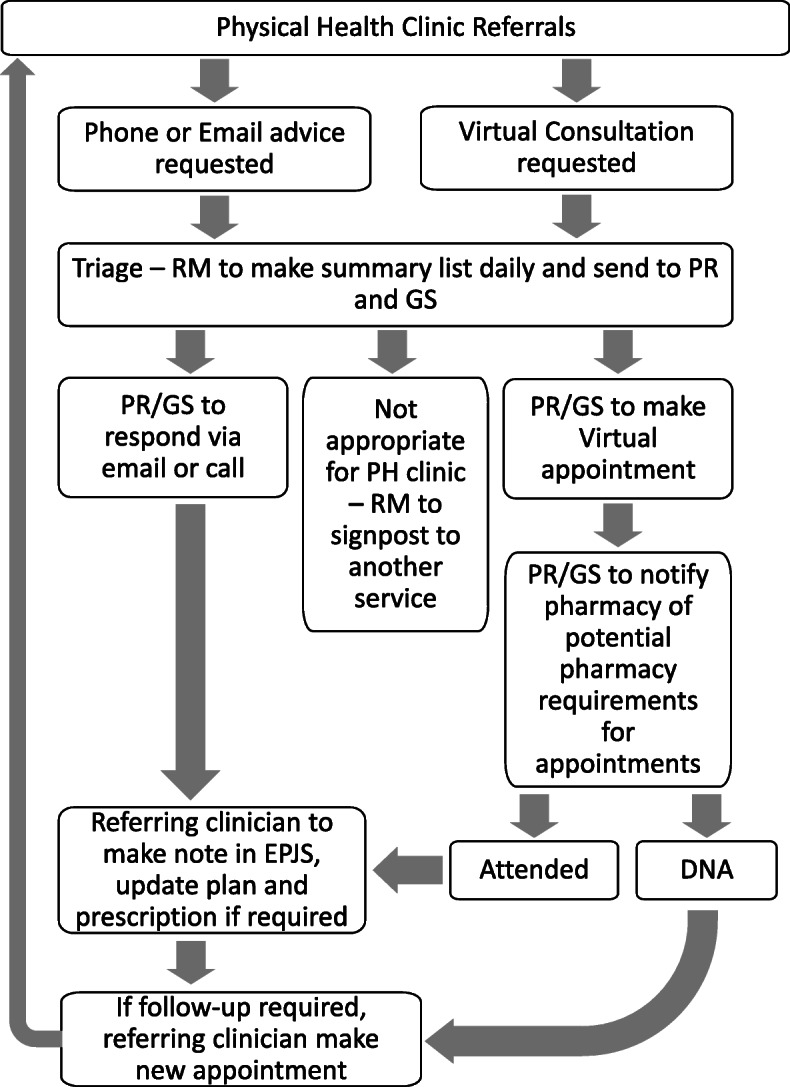
Virtual Physical Health clinic flowchart

The process for the VPHC is set out in Fig. 2.

#### CCc

CC can be accessed via an app downloaded on to either a mobile phone or iPad or by calling the CC telephone number. We will encourage staff to use the app as it has better usability for referrers and allows data collection which is not possible when using a telephone call. All wards have an iPad and connectivity will be tested before going live. There may be potential limitations for the use of the mobile app where Internet connection is poor or clinicians may not want or be able to download the app to their personal mobile.

A new ‘note type’ has been added to the existing documenting options within ePJS ‘Ward Progress Note’ and ‘Event’ for staff to document the conversations and advice given during CC calls.

SLaM staff will be made aware of the VPHC and CC roll-out using a range of communications, including:

- Trust communication department
- Emails to: consultants, medical trainees, pharmacists, inpatient ward managers, heads of nursing and quality and modern matrons
- Link on SLaM intranet physical health page
- Presentation at the Trust Physical Health Strategy board, junior doctor inductions and relevant consultant (attending-level physicians) meetings
- Trainee doctor WhatsApp group

Information will be provided on both services with clear information on which service to refer to.

### Evaluation review process and approvals

The evaluation protocol will be submitted for institutional review and approval prior to data collection. CRIS data approval will be sought from the CRIS Oversight Board. Routinely collected data approval will be sought as a service redesign evaluation. For self-reported data, which will require surveys to be administered to SLaM staff, we will seek SLaM clinical governance approval.

### Evaluation aim 1: assessment and optimisation of implementation strategies

The evaluation team will map the different implementation strategies applied to both the VPHC and the CC service rollout using the Expert Recommendations for Implementing Change (ERIC) implementation strategies framework, which has identified 73 discrete implementation strategies based on a systematic review of the healthcare evidence base and expert consensus [12]. We will assess how many of these strategies have been used in the implementation of the two services, how the strategy was put in place and whether different strategies have (or could be, based on uptake data and user feedback) been applied at different time points. These will be reviewed for efficacy regularly on a pragmatic basis, in light of the uptake data available to the evaluation team; changes to implementation strategies over time and rationale for such changes will be recorded for subsequent analysis.

### Aim 2: assessment of acceptability and feasibility

The *primary implementation outcome* to be assessed is the uptake of each of the two interventions. VPHC data will be collated weekly, and CC data will be collated monthly.

*VPHC*: the primary outcome will be the number of referrals made. This will be monitored via the ePJS form and a database designed by the IMPHS team to document referrals. This form will include details of referrer, ward and purpose of the referral.

*CC*: the primary outcome will be the number of calls made*.* These data will be derived from routinely collected CC data.

### Secondary outcomes

Specific outcomes for the VPHC (all routinely recorded):

1. Number of appointments booked that take place.
2. Reasons given for appointments not attended and any alternative actions taken.
3. Time spent on triage, telephone advice and virtual appointments by the VPHC consultant and ANP.

Specific outcomes for CC (all routinely recorded): referral avoided, referral made, admission avoided, admission made, diagnostics requested.

The CC system asks referrers to document the actions that are taken from the CC call. It should be noted that this is only recorded if the referrer stays on the line after the call to complete this and currently take-up of this is low. If the CC app is used the name of referrer, speciality called, length of time waiting to be connected and length of call is also documented.

*Tertiary outcomes* (for both VPHC and CC, to be collected via self-report):

We will collect feedback from staff on the usability of both services using a questionnaire administered either online or by telephone. The questionnaire will include questions on the following: experiences of using either VPHC or CC, whether using either service was perceived to decrease the time spent on managing physical health compared with previous experiences, any perceived barriers to using either service and any unintended consequences for the patient or the ward in using either service. Participants will also be asked to complete the validated Acceptability Intervention Measure (4 questions), Intervention Appropriateness Measure (4 questions) and Feasibility of Intervention Measure (4 questions) [13]. These measures will offer a standardised assessment of three well-established facets of implementation success, namely how acceptable, appropriate and feasible a new intervention and the manner in which it is being implemented is seen by its intended users [14]. We will consider the feasibility of collecting these scales at regular intervals (e.g. 4-monthly) to allow us to assess longitudinal fluctuations in overall ‘implementability’ of the two interventions as the strategies to support implementation are optimised through the study.

### Implementation data analysis

Contextual factors will be documented throughout roll out such as ongoing changes to SLaM ward configurations in response to infection prevention and control guidelines or any changes to services provided by local Acute Trusts. This contextual data will be used to understand external influences on the uptake of the VPHC and CC and the running of the VPHC.

For our primary implementation assessment, data on implementation strategies applied will be collected and a descriptive analysis conducted, categorised by service (VPHC and CC). The number and type of strategies used will be recorded. Precisely how the strategies were implemented will be described qualitatively and any shifts in strategies over time also recorded.

For our secondary implementation assessment, a descriptive analysis will be conducted for both services for the primary (measured as number of referrals being made for VPHC on a monthly basis and number of calls being made for CC on a weekly basis), secondary (measured as number of appointments, reasons of appointments and access to internal triage) and implementation outcomes. In order to monitor primary and secondary outcomes, descriptive graphs and Statistical Process Control charts will be employed on weekly and monthly basis.

For our tertiary implementation assessment, standardised implementation survey outcomes (AIM, IAM, FIM) will be assessed via a descriptive statistical analysis. Difference in the total scores of implementation survey outcomes by service (VPHC vs CC) and within each service over time (to capture any effects of different implementation strategies) will be assessed with the use of generalised regression models (Poisson or linear depending on the distribution of the outcome).

As the evaluation design is formative, outcomes will be analysed and fed back to SLaM via the Physical Health Implementation Committee and the IMPHS steering group, and this will be provided in an easily understood usage report. This will allow adjustments to be made to the implementation process of both interventions.

### Aim 3: informing future roll out and further impact evaluation

The rapid evaluation phase will be used to cost the intervention and the resource involved in implementation and provide indicative information on whether the intervention may be cost saving.

#### Cost comparison scenario analysis

Information on baseline healthcare utilisation will inform estimates of the overall risk of A&E attendances/admissions. Threshold analysis will compare the estimated cost with the required effect the interventions would need to have on healthcare utilisation in order for the interventions to be cost neutral or cost saving. The method for estimating costs of the interventions and the implementation is outlined below.

#### Quantifying resource use associated with the intervention and implementation:

Resource inputs required to set up, administrate and run the VPHC including any staff training undertaken will be estimated in consultation with IMPHS project team and VPHC clinicians who will maintain a resource use log throughout the set up and roll out.

#### Quantifying impact on clinical time

Time spent making referrals and calls to VPHC and CC will be quantified. For VPHC, this time will be recorded on ePJS. For CC, the time spent waiting for a call to be answered and the length of the call is recorded routinely.

Estimates will also be made of the impact on Maudsley staff time through a baseline and follow-up survey of ward staff to identify any changes in time spent undertaking administrative tasks associated with physical healthcare management.

#### Quantifying impact on wider healthcare utilisation

Data will be analysed from available datasets to establish a baseline level of healthcare utilisation to inform the cost comparison scenario analysis. We will collect data from the year before the VPHC and CC begin and during the pandemic. The following data will be evaluated for baseline estimation and ongoing monitoring:

1. Number of referrals/attendances to Emergency Departments (ED) or other Acute Trust services per month before and during the roll out.
2. Length of stay in ED or Acute Trust wards before and during roll out.
3. Length of stay on SLaM wards before and during roll out.

#### Valuing resource use

Resource use will be valued using national unit cost data for hospital staff or locally derived staff costs where possible. This will generate cost estimates of the two interventions, and implementation costs, from an NHS provider perspective.

#### Establishing anticipated effects beyond healthcare utilisation including patient benefit

Given the complexity of the two interventions and scarcity of evidence on likely effect, an exploratory analysis phase is required. The two interventions are complex and as they are not targeted to one clinical area the ramifications are likely to be diffuse. The initial evaluation phase will therefore be used to monitor potential ‘spill over effects’ (unintended positive or negative consequences) and establish evidence for the likely patient benefit. In line with the draft Medical Research Council (MRC) guidelines for evaluating complex interventions [15], the mixed methods approach will help establish the expected causal mechanism for the intervention.

Drawing on Yao and colleague’s [16] framework for evaluating service delivery interventions at the scoping stage, the first phase implementation will be used to:

- Understand the anticipated area of patient benefit by profiling the reasons for referrals and contacts with the VPHC and CC
- Identify and categorise the multiple outcomes associated with the interventions that are measurable and the data sources for these. Outcomes will include adverse events such as hospital admission/A&E attendances as outlined above. Measurable outcomes may also include patient physical health safety incidences, mortality and clinical effectiveness metrics, e.g. HbA1c control.
- Investigate and measure important positive and negative unintended consequences such as the uptake of physical healthcare monitoring in line with the NICE concordant annual health check for people with severe mental illness).
- Investigate the feasibility of estimating the utility associated with the measurable outcomes using available literature. Utility estimates may then be used in further economic evaluation.
- Estimate baseline and follow-up disease prevalence rates and estimate levels of unmet need to determine plausible effect estimates and to form future hypothesis for longer term evaluation.

The evidence generated will be used in a series of virtual workshops with project team, VPHC staff and ward staff to produce a logic model to conceptualise the decision problem and agree the cost and outcome variables that should be used in further evaluation and therefore inform ongoing measurement requirements.

## Discussion

This pragmatic evaluation aims to understand how two new services are implemented, their impact on the management of physical health in SLaM inpatient units and the potential economic impact of the services by estimating the level of effect on healthcare utilisation required for the services to be cost neutral or cost saving.

The study is subject to limitations associated with its design, which in turn is affected by the ongoing COVID-19 pandemic and associated clinical needs. The evaluation is pragmatic such that it is designed around services as delivered rather than as optimised for a gold standard randomised evaluation. This means that we lack control groups and the usual separation between the implementation and evaluation teams. These would be both unachievable in the context of the services which will be evaluated and undesirable given the logistical complexity, length (hence time delay to reveal findings) and resources that a fully controlled, external, ‘summative’ evaluation would incur. These limitations are inherent to this type of evaluation and we remain conscious of their impact on our ability to draw firm conclusions. Causal attributions in terms of clinical effectiveness of either service or the effectiveness of different implementation strategies in getting the interventions sustainably applied fall outside the remit of this study.

The study also has strengths. As a formative evaluation, it has been designed jointly with stakeholders from its inception, hence the aims of the study, measures to be collected and analyses to be carried out are of direct relevance to SLaM service leads, frontline staff and managers and will be used within the Trust hospitals when available. Currently, there is little literature on the evidence for this type of integration of services in this way [17], so this study will provide useful information on this type of integration which is particularly timely during the COVID-19 pandemic with the associated changes made to services. The expected benefits of these services are improved management of physical health conditions on psychiatric inpatients units and ultimately better physical health outcomes for service users. Whilst further studies need to be done to determine such clinical impacts, the present study will facilitate understanding of how to implement such service innovations in the first instance.

## Data Availability

N/a.

## Abbreviations

SMI: Severe mental illness
VPHC: Virtual Physical Health Clinic
CC: Consultant Connect
NICE: National Institute for Health and Care Excellence
IMPHS: Integrating our Mental and Physical Health Care Systems
SLaM: South London and Maudsley NHS Foundation Trust
KHP: King’s Health Partners
BRC: Biomedical Research Centre
CRIS: Clinical Record Interactive Research System
ePJS: Electronic Patient Journey System
HES: Hospital Episodes Statistics
NHS: National Health Service
ED: Emergency Department
